# Molecular Genetic and Genomic Alterations in Cushing’s Syndrome and Primary Aldosteronism

**DOI:** 10.3389/fendo.2021.632543

**Published:** 2021-03-12

**Authors:** Crystal D. C. Kamilaris, Constantine A. Stratakis, Fady Hannah-Shmouni

**Affiliations:** Section on Endocrinology and Genetics, Eunice Kennedy Shriver National Institute of Child Health & Human Development (NICHD), National Institutes of Health (NIH), Bethesda, MD, United States

**Keywords:** Cushing’s syndrome, genetics, primary aldosteronism, adrenocortical hyperplasia, adrenocortical adenoma

## Abstract

The genetic alterations that cause the development of glucocorticoid and/or mineralocorticoid producing benign adrenocortical tumors and hyperplasias have largely been elucidated over the past two decades through advances in genomics. In benign aldosterone-producing adrenocortical tumors and hyperplasias, alteration of intracellular calcium signaling has been found to be significant in aldosterone hypersecretion, with causative defects including those in *KCNJ5, ATP1A1, ATP2B3, CACNA1D, CACNA1H*, and *CLCN2.* In benign cortisol-producing adrenocortical tumors and hyperplasias abnormal cyclic adenosine monophosphate-protein kinase A signaling has been found to play a central role in tumorigenesis, with pathogenic variants in *GNAS, PRKAR1A, PRKACA, PRKACB, PDE11A*, and *PDE8B* being implicated. The role of this signaling pathway in the development of Cushing’s syndrome and adrenocortical tumors was initially discovered through the study of the underlying genetic defects causing the rare multiple endocrine neoplasia syndromes McCune-Albright syndrome and Carney complex with subsequent identification of defects in genes affecting the cyclic adenosine monophosphate-protein kinase A pathway in sporadic tumors. Additionally, germline pathogenic variants in *ARMC5*, a putative tumor suppressor, were found to be a cause of cortisol-producing primary bilateral macronodular adrenal hyperplasia. This review describes the genetic causes of benign cortisol- and aldosterone-producing adrenocortical tumors.

## Introduction

The discovery of the genetic drivers of adrenocortical tumorigenesis has been facilitated by advances in genomics over recent years and has provided new insights on the molecular pathogenesis of adrenocortical disease. The identification of disease-causing germline and somatic pathogenic variants in primary aldosteronism (PA) and Cushing’s syndrome (CS) is ushering in a new era of “precision-medicine,” though the challenge in future years is to translate these discoveries into new diagnostic and therapeutic modalities. Furthermore, these discoveries have allowed for a more specific classification of adrenocortical hyperplasias, that goes beyond pathology and is gene-based, allowing for more patient-specific genetic screening and counseling. In PA, these discoveries include the identification of the crucial role of aberrant intracellular calcium signaling in aldosterone hypersecretion, with defects in genes that encode ion channels, including *KCNJ5, CLCN2, CACNA1H* and *CACNA1D*, and ATPases, such as *ATP1A1* and *ATP2B3*, being implicated in this pathogenesis. In CS abnormal cyclic adenosine monophosphate (cAMP)-protein kinase A (PKA) signaling has been implicated in the development of most benign cortisol-producing adrenocortical tumors and hyperplasias (1–4). Aberrant cAMP-PKA signaling was first associated with the development CS due to primary bimorphic adrenocortical disease (PBAD) in the rare tumor disorder McCune-Albright syndrome (MAS), which is caused by early embryonic postzygotic somatic activating defects in *GNAS*, the gene that encodes the α-subunit of the stimulatory G protein (G_s_α) (2, 4). This pathway was further implicated in the development of CS due to primary pigmented nodular adrenocortical disease (PPNAD) through the study of another rare familial tumor syndrome, Carney complex (CNC), that is predominantly due to germline inactivating defects in *PRKAR1A*, which encodes the regulatory subunit type 1α (R1α) of PKA (3). Subsequently aberrant cAMP-PKA signaling was identified as a significant cause of cortisol-producing adrenocortical adenomas (CPAs) through activating somatic defects in *PRKACA*, the gene that encodes the catalytic subunit Cα of PKA (1). In bilateral macronodular adrenal hyperplasia (PBMAH), another form of adrenocortical hyperplasia, germline defects in the tumor suppressor gene *ARMC5* were found to be the most common underlying genetic defect (5).

Primary adrenocortical tumors (ACTs) are primarily comprised of benign adenomas and/or hyperplasias and less frequently carcinoma. These tumors may be sporadic or familial, unilateral or bilateral, and secreting or non-secreting. In a retrospective population-based cohort study that evaluated the standardized incidence rate of adrenal tumors in all patients with tumors who lived in Olmsted County, Minnesota, USA from 1995 to 2017, the overall mean sex-standardized and age-standardized incidence rates of adrenal tumors diagnosed from 1995 to 2017 was 47 (95% CI 45–50) per 100,000 person-years, with the incidence of adrenal tumors increasing ten times from 1995 to 2017 paralleling the increased use of abdominal imaging (6). The prevalence of adrenal tumors in 2017 was 0.53%. Of the 1287 patients identified as having adrenal tumors, 93.7% had adrenocortical adenoma and nodular hyperplasia, 8.6% had malignant masses, 6.6% had other benign masses, and 1.1% had pheochromocytoma, with 4.1% having overt adrenocortical hormone excess. This review focuses on the reported causative genomic alterations in benign cortisol- and/or aldosterone-producing ACTs.

## Aldosterone-Producing Adrenocortical Tumors

PA is the most common cause of secondary hypertension, and is responsible for approximately 8% of cases. It is characterized by aldosterone secretion that is relatively autonomous of the major regulators of secretion and inappropriately high for sodium status, and is not suppressed by sodium loading. This results in sodium retention, suppression of plasma renin, hypertension, and increased potassium excretion (7–9). Bilateral adrenocortical hyperplasia (BAH) and aldosterone-producing adenomas (APAs) account for 65% and 35% of cases of PA, respectively, with less common causes including unilateral hyperplasia (2%), pure aldosterone-producing ACC (<1%), familial hyperaldosteronism (FH) (FH type 1 <1%), and ectopic aldosterone-producing adenoma or carcinoma (<0.1%) (9). Intravascular volume depletion and elevation in plasma potassium are the main stimuli for aldosterone synthesis. Intravascular volume depletion causes activation of the renin-angiotensin system with release of angiotensin II (AT-II) which binds to a G-protein coupled receptor (GPCR) on the adrenocortical zona glomerulosa cells, while increased potassium directly raises the production of aldosterone in adrenocortical zona glomerulosa cells, whose resting potential is set by potassium channel activity (10). These physiologic stimuli exert their effects through the generation of a cytoplasmic calcium signal through membrane depolarization with activation of voltage-gated calcium channels and increased intracellular calcium. This leads to increased expression of aldosterone synthase (CYP11B2), increased aldosterone production, and glomerulosa cell proliferation. Aldosterone acts on the mineralocorticoid receptor in the renal distal convoluted tubule, connecting tubule, and cortical collecting duct, amongst other tissues, with resulting increased renal sodium reabsorption and potassium excretion.

### Familial Hyperaldosteronism

FH is inherited in an autosomal dominant manner. Four major types of FH, type I through IV, have been described, with FH comprising 1% to 5% of PA cases. FH type I (FH-I) or glucocorticoid remediable hyperaldosteronism (GRA) is the result of aldosterone overproduction due to ACTH-dependent activation of aldosterone synthase and was initially described in 1966 in a single family, with identification of the causative chimeric gene 26 years later (11, 12). GRA is the result of the formation of a chimeric gene from unequal crossing over between 2 highly homologous genes that encode isozymes of 11-beta-hydroxylase on chromosome 8: *CYP11B1*, which encodes 11β-hydroxylase (catalyzes conversion of 11-deoxycortisol to cortisol), and *CYP11B2*, which encodes aldosterone synthase (converts deoxycorticosterone to corticosterone and 18-hydroxycorticosterone to aldosterone). The fusion of the promoter region of *CYP11B1* with *CYP11B2*, leads to ectopic expression of *CYP11B2* in the zona fasciculata with ACTH-dependent activation of the aldosterone synthase. This condition can be treated with intermediate-acting glucocorticoids administered at bedtime at the smallest effective dose, with or without mineralocorticoid antagonist therapy (13). Glucocorticoids diminish ACTH release and can reverse the hypersecretion of aldosterone. FH type II (FH-II) was first described in 1992 as familial PA due to APA and/or BAH without response to glucocorticoid administration and did not have a known genetic etiology until recently. Initially, in the year 2000, a locus for FH-II was identified on the short arm of chromosome 7 corresponding to the band 7p22 (14). Subsequently it was discovered that gain-of-function defects in the *CLCN2* gene, which encodes the chloride channel ClC-2, were the causative pathogenic variants in a subset of patients (15). These *CLCN2* defects cause voltage-gated calcium influx due to increased chloride permeability and depolarization (15, 16). A study that included a family with FH-II and 80 additional probands with unsolved early-onset PA initially identified defects in *CLCN2* as the cause of FH-II, with eight probands carrying heterozygous variants in *CLCN2*, including two *de novo* defects and all relatives with early-onset PA harboring the *CLCN2* variant found in the probands (16). FH type III (FH-III) is the result of germline defects in the *KCNJ5* gene, and was first described in 2008 in a family presenting with a novel form of glucocorticoid-refractory PA (17). *KCNJ5* encodes GIRK4 (G-protein–activated inward rectifier potassium channel 4), an inwardly rectifying potassium channel. Defects in this gene result in altered channel selectivity that causes increased sodium conductance and cell depolarization, ultimately leading to increased intracellular calcium and calcium signaling (17). A case of early-onset PA with BAH caused by mosaicism for a *KCNJ5* defect was also recently described (18). Somatic defects in *KCNJ5* are the most common genetic defect associated with APAs, with APA-causing somatic pathogenic *KCNJ5* variants leading to a more severe phenotype when found in the germline, as opposed to *KCNJ5* defects identified only in the germline, which tend to lead to a milder phenotype, though there are some exceptions. FH type IV is the result of germline defects in the *CACNA1H* gene and was first described in 2015 (19). *CACNA1H* encodes a T-type calcium channel, with pathogenic variants in this gene causing increased intracellular calcium through impaired channel inactivation and activation at more hyperpolarized potentials (19). Germline *CACNA1H* defects were initially identified in five unrelated individuals with early-onset PA with family analysis being suggestive of incomplete penetrance and showing *de novo* occurrence in two kindreds. Germline defects in *CACNA1D*, which encodes an L-type calcium channel, cause early-onset PA, seizures, and neurologic abnormalities, referred to as PA with seizures and neurological abnormalities or PASNA syndrome (20). Mutant channels are activated at less depolarized membrane potentials and show impaired inactivation, with resulting increased calcium influx. Due to the severity of the associated disease, these variants occur exclusively *de novo* and are not inherited (20). Germline *ARMC5* pathogenic variants have also been associated with PA and germline variants in the phosphodiesterase 2A (*PDE2A*) and 3B (*PDE3B*) genes, were recently associated with PA caused by BAH, however these genetic defects have not yet been designated as causes of FH (21, 22). In one study that included 56 subjects with PA, some of whom had BAH, six subjects (10.7%) harbored a germline *ARMC5* variant that was predicted to be pathogenic by in silico analysis, with all six of these subjects being African American (22). However, this was not confirmed in a subsequent study of 39 primarily Caucasian patients (37 Caucasian and 2 African American subjects) with PA and BAH, where no germline pathogenic *ARMC5* variants were identified (23).

### Aldosterone-Producing Adrenocortical Adenomas

**Figure 1:**
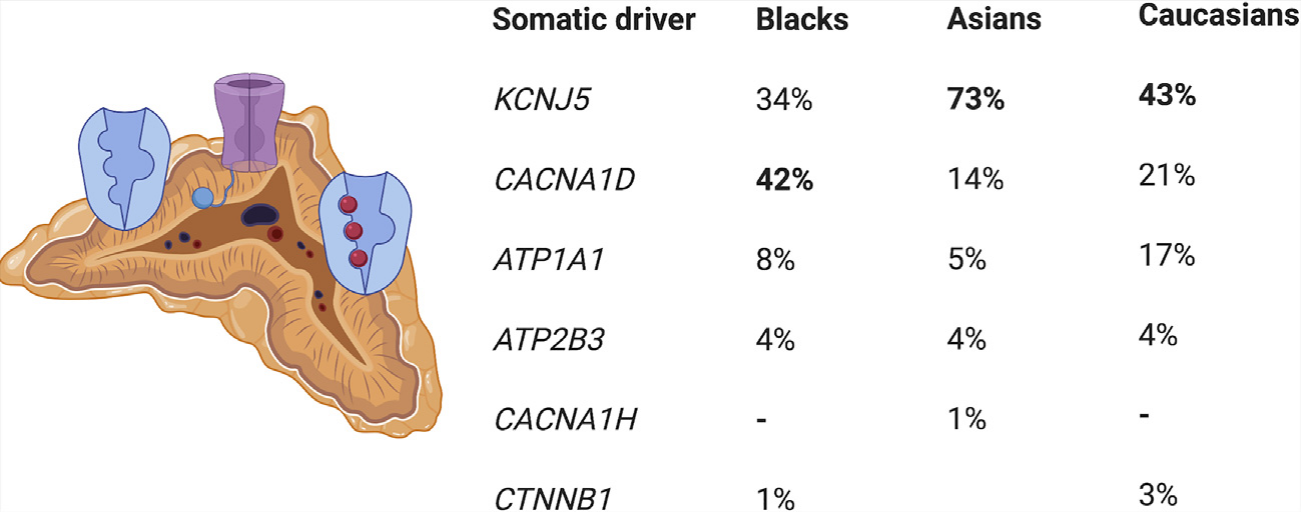
Prevalence of somatic driver pathogenic variants in aldosterone-producing adenomas. KCNJ5, potassium channel, inwardly rectifying, subfamily J, member 5; CACNA1D, calcium channel, voltage dependent, L-type, alpha-1D subunit; ATP1A1 ATPase, NA+/K+ transporting, alpha-1 polypeptide,; ATP2B3, ATPase, Ca(2+)-transporting, plasma membrane, 3; CACNA1H, calcium channel, voltage dependent, T-type, alpha-1H subunit; CTNNBI, catenin, beta-1.

**Figure 2:**
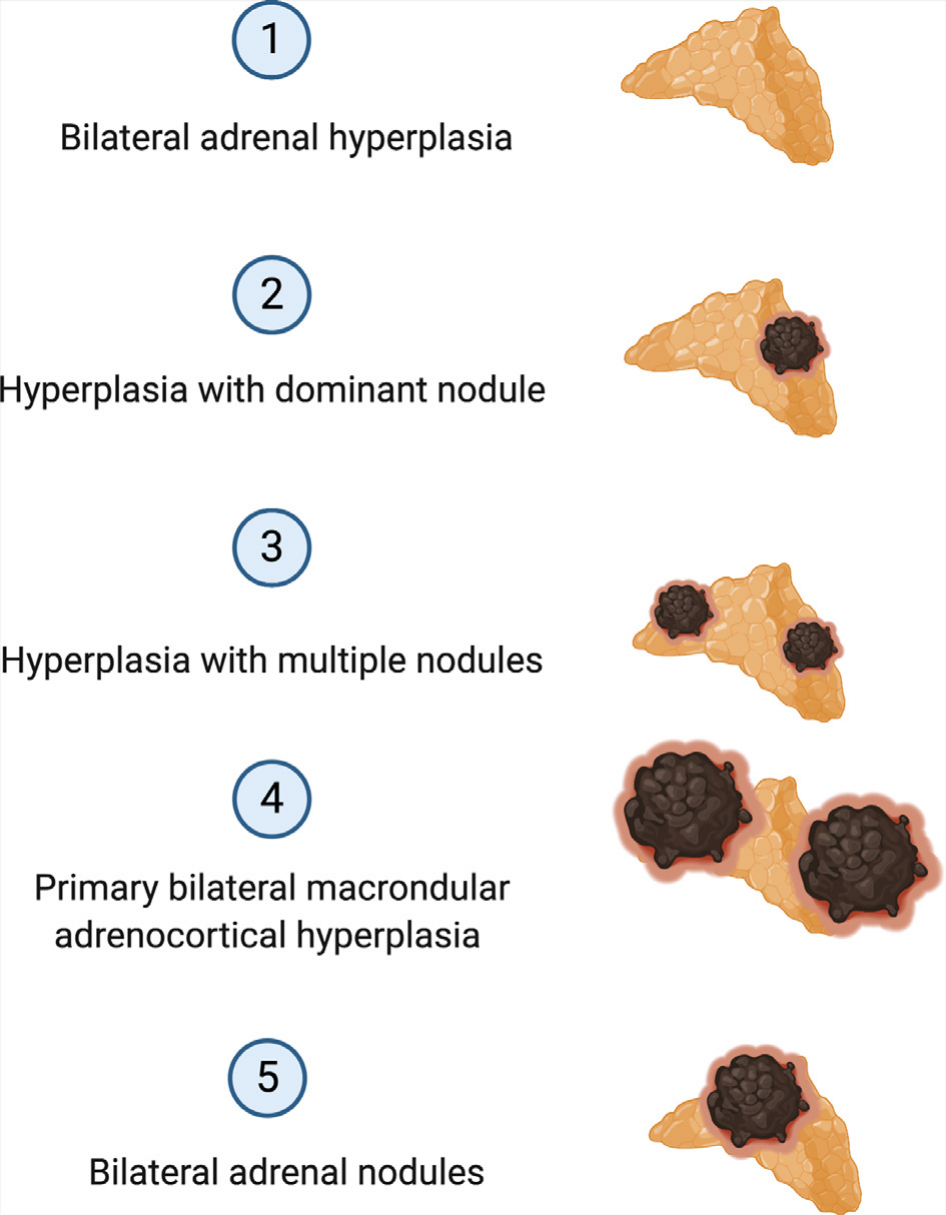
Histologic Classification of Primary Aldosteronism.

Approximately 90% of APAs are caused by somatic pathogenic variants in genes encoding ion channels or transporters, such as those in *KCNJ5, CACNA1D, ATP1A1* and *ATP2B3* (20, 24–26) (**Figure 1**). Somatic variants in *KCNJ5* are associated with 40% of APAs, with two hotspots, p.G151R and p.L168R, in and near the selectivity filter of KCNJ5 being responsible for the majority of *KCNJ5* defects in APAs. Defects in *KCNJ5* lead to more severe PA at a younger age, with larger APAs, and are more common in females than in males (53–63% vs. 22–31%), with a higher frequency in some Asian cohorts (60–70% of APAs) compared to European cohorts (26–31) (**Figure 1**). Somatic defects in *CACNA1D* account for 21% to 42% of APAs, making this the second most common defect in these tumors (32). In contrast to patients of European and Asian decent where somatic *KCNJ5* defects are the most prevalent APA-causing genetic alterations, in Blacks, somatic *CACNA1D* defects were found to be the most prevalent genetic defect in APAs (**Figure 1**) (29–31, 33). Defects in *CACNA1D* were more frequent in APAs from Black males as opposed to Black females, who unlike males still have a high rate of *KCNJ5* pathogenic variants (33). Forty-two percent of APAs in Blacks harbored *CACNA1D* defects, followed by *KCNJ5* in 34%, *ATP1A1* in 8%, and *ATP2B3* in 4% (33). Three to 17% of APAs result from gain of function somatic pathogenic variants in the ATPase genes *ATP1A1* and *ATP2B3* (25, 32). Defects in these genes result in abnormal Na+ or H+ permeability and increased aldosterone production. The wingless-type (*Wnt*)–β-catenin pathway has also been associated with APA formation, with 2% to 5% of APAs harboring activating somatic pathogenic variants in the β-catenin gene *CTNNB1* and a significant proportion of APAs demonstrating constitutive activation of the Wnt-β-catenin pathway (20, 34–37). The cytoplasmic protein β-catenin is the main player in this pathway. Its stability is regulated by the Axin complex which is comprised of the scaffolding protein Axin, the tumor suppressor *adenomatous polyposis coli* (APC) gene product, casein kinase 1 (CK1) and glycogen synthase kinase 3 (GSK3). When Wnt is absent, β-catenin is continually degraded by the action of the Axin complex. This does not allow β-catenin to translocate to the nucleus, with resulting repression of Wnt target genes by the DNA-bound T cell factor/lymphoid enhancer factor (TCF/LEF) family of proteins. Binding of Wnt ligands to a receptor complex including a member of the frizzled family of seven-transmembrane receptors and a member of the LDL receptor family (LRP 5 or 6) activates the Wnt-β-catenin pathway, with subsequent inhibition of the Axin complex, and stabilization and accumulation of β-catenin. Beta-catenin then translocates to the nucleus where it activates TCF/LEF transcription factors thereby activating Wnt target gene expression (38). Two women with APAs that presented in pregnancy were also found to have somatic pathogenic variants in *CTNNB1* and had significantly upregulated adrenocortical expression of the LH/hCG receptor and gonadotropin releasing hormone (GnRH) receptor (39). However, a subsequent study that genotyped subjects with PA and evaluated *in vivo* for GnRH/LH responsive aldosterone secretion, found that aberrant aldosterone regulation occurred frequently in PA, but was not often associated with *CTNNB1* pathogenic variants (40). Recently somatic *CACNA1H* pathogenic variants were identified as a cause of APAs, however the prevalence of these pathogenic variants in APAs is low (41). Somatic pathogenic variants in *CLCN2* were also recently described in APAs with a prevalence of 1.74% in one small study (42). The histologic classification of PA is a spectrum, ranging from BAH to PBMAH, as shown in **Figure 2** (43).

## Cortisol-Producing Adrenocortical Tumors

**Figure 3:**
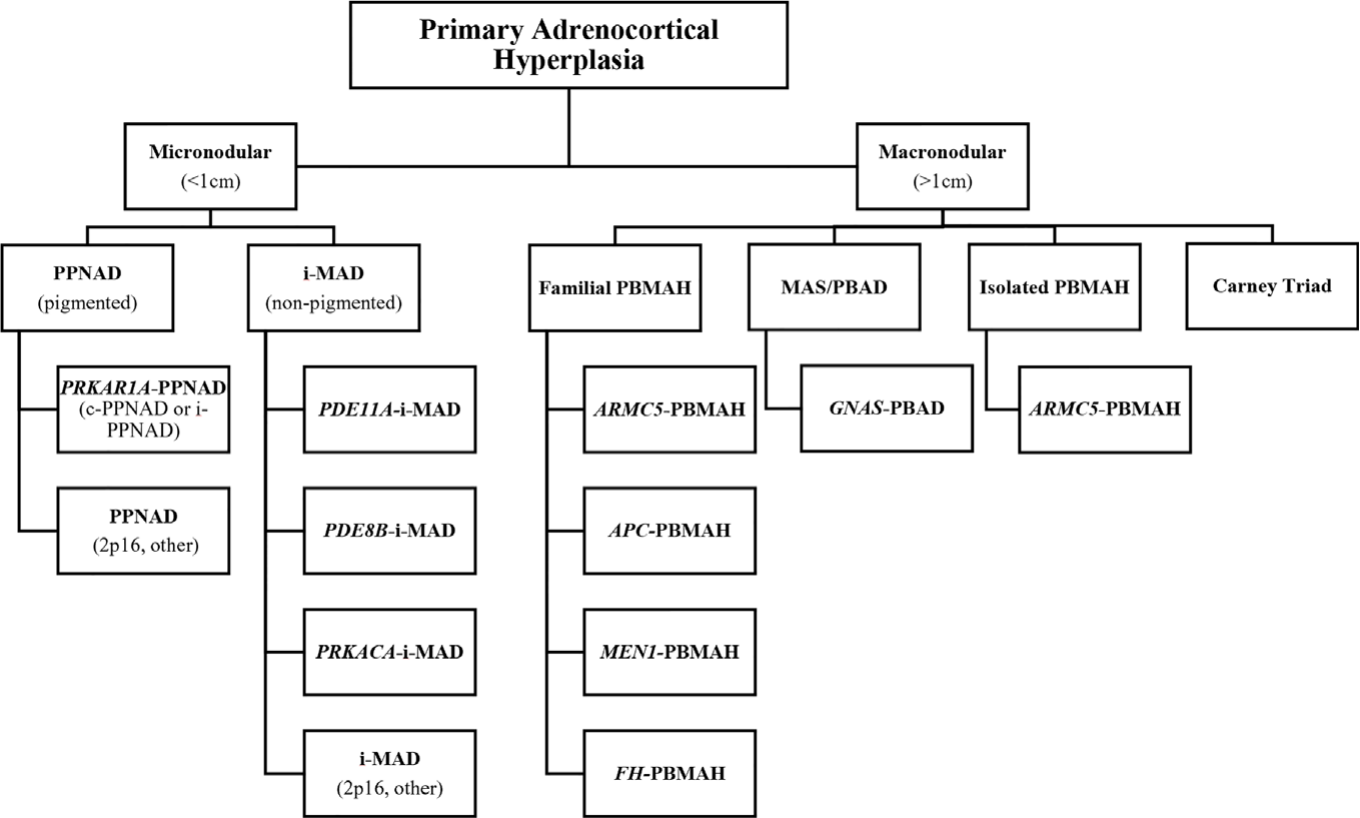
Gene-based diagnostic algorithm for primary cortisol-producing adrenocortical hyperplasias. *APC, adenomatous polyposis coli gene; ARMC5, armadillo repeat-containing protein 5; c-PPNAD, CNC-associated primary pigmented nodular adrenocortical disease; CNC, Carney complex; FH, fumarate hydratase; GNAS, gene coding for the stimulatory subunit α of the G-protein (Gsα); i-MAD, isolated micronodular adrenocortical disease; i-PPNAD, isolated PPNAD; MAS, McCune–Albright syndrome; MEN1, multiple endocrine neoplasia type 1; PBAD, primary bimorphic adrenocortical disease; PBMAH, primary bilateral macronodular adrenocortical hyperplasia; PDE8B, phosphodiesterase 8B gene; PDE11A, phosphodiesterase 11A gene PPNAD, primary pigmented nodular adrenocortical disease; PRKACA, protein kinase, cAMP-dependent, catalytic, alpha; PRKAR1A, protein kinase, cAMP-dependent, regulatory, type I, α gene. *Adapted from Kamilaris CDC, Stratakis CA, Hannah-Shmouni F: Adrenocortical tumorigenesis: Lessons from genetics. Best Practice & Research Clinical Endocrinology & Metabolism 2020,34(3):101428.

CS has an incidence of two to three cases per one million inhabitants per year, and is comprised of signs and symptoms that develop due to prolonged tissue exposure to excess glucocorticoids, with approximately 20% of cases of endogenous CS being caused by primary adrenocortical hyperfunction (10–22% CPAs, 1–2% adrenocortical hyperplasia which is mostly bilateral, and 5–7% adrenocortical carcinoma [ACC]) (44–46). Specifically, BAH is characterized by multiple adrenocortical nodules and is classified as micronodular or macronodular depending on whether the radiological size of most of the nodules is less than or greater than 1 cm in diameter, respectively. BAH is further subclassified histologically according to the histological presence of pigment (lipofuscin) inside the nodules or in the surrounding adrenal cortex and/or the presence of atrophy or hyperplasia of the internodular cortex. The bilateral nature of the ACTs in BAH suggests an underlying genetic predisposition which has been confirmed in many cases, and a gene-based subclassification was recently proposed (**Table 1** and **Figure 3**) (47, 48).

**Table 1:** Classification and characteristics of primary cortisol-producing adrenocortical hyperplasia.*

Adrenocortical lesions	Genes (locus)	Histopathology	Characteristics
PBMAH	*ARMC5* (16p11.2) *MEN1* (11q13) *FH* (1q42.3-43) *APC* (5q22.2) *PDE11A* (2q31.2) *PDE8B* (5q13.3) *GNAS* (20q13) *PRKACA* duplication (19p13.1)	-Nodules or macroadenomatous, > 1 cm, with (type 1) or without (type 2) internodular atrophy. -Hyperplasia with dominant nodule.	-Middle age, mild hypercortisolism and/or mineralocorticoid excess -Associated with MEN-1, FAP, MAS, HLRCC, isolated (AD) -Most lesions have aberrant GPCRs (vasopressin, serotonin, catecholamines, GIP, luteinizing hormone) -PBMAH carry the ability of intra-adrenal production of ACTH with an autocrine/paracrine effect on glucocorticoid or mineralocorticoid production
PBAD	*GNAS* (20q13; mosaic)	-Distinct adenomas (>1cm), with occasional microadenomas and internodular atrophy	-Infants and very young children -MAS
FDCS (GIP-dependent)	*GIPR* gene (19q13.32) duplication	-Large adenomas and/or macronodules	-Aberrant GPCRs (GIPR) -Low fasting cortisol, hypercortisolism post-meals
i-PPNAD	*PRKAR1A* (17q22-24) *PRKACA* duplications (19p13.1)	-Microadenomatous (<1cm) hyperplasia with pigmentation	-Children and young adults -Lentiginosis in few cases
c-PPNAD	*PRKAR1A* (17q22-24, CNC1 locus) 2p16 (CNC2 locus, unknown gene)	-Microadenomatous (<1cm) hyperplasia with (mostly) internodular atrophy and pigmentation	-Children, young and middle aged adults -Disease at a younger age and a higher frequency of myxomas, schwannomas, and thyroid and gonadal tumors than patients without *PRKAR1A* variants. -In-frame deletion of exon 3 and the c.708 +1G>T appears to confer a more severe CNC phenotype, while the splice variant c.709(-7-2)del6 and the initiation alternating substitution c.1A>G/p.M1Vp has been associated with incomplete penetrance of CNC, as seen in i-PPNAD -CNC1: The hot spot c.491-492delTG is most closely associated with lentigines, cardiac myxoma, and thyroid tumors when opposed to all other *PRKAR1A* variants -Expressed RIα mutant protein present with more severe and aggressive CNC-phenotype -CNC2: Sporadic disease later in life with a lower frequency of myxomas, schwannomas, thyroid tumors, and LCCSCT
i-MAD	*PDE11A* (2q31.2) *PDE8B* (5q13) *PRKACA* (19p13.1) 2p16 (unknown gene)	-Microadenomatous (<1cm) hyperplasia with internodular hyperplasia and limited or absent pigmentation	-Mostly children and young adults -Cyclical hypercortisolism -May be associated with a paradoxical rise of glucocorticoid excretion during the Liddle’s test -Isolated or AD

APC, adenomatous polyposis coli gene; c-PPNAD, CNC-associated primary pigmented nodular adrenocortical disease; CNC, Carney complex; FAP, familial adenomatous polyposis; FDCS, food-dependent Cushing’s syndrome; GNAS, gene coding for the stimulatory subunit α of the G-protein (Gsα); GPCR, G-protein-coupled receptor; HLRCC, hereditary leiomyomatosis and renal cancer syndrome; i-MAD, isolated micronodular adrenocortical disease; i-PPNAD, isolated PPNAD; LCCSCT, large cell calcifying Sertoli cell tumor; MAS, McCune–Albright syndrome; MEN1, multiple endocrine neoplasia type 1; PBAD, primary bimorphic adrenocortical disease; PBMAH, primary bilateral macronodular adrenocortical hyperplasia; PDE8B, phosphodiesterase 8B gene; PDE11A, phosphodiesterase 11A gene; PRKAR1A, protein kinase, cAMP-dependent, regulatory, type I, α gene.

*Adapted from Hannah-Shmouni F, Stratakis CA: A Gene-Based Classification of Primary Adrenocortical Hyperplasias. Hormone and Metabolic Research 2020, 52(3):133-141.

The cAMP-PKA pathway is central to the regulation of adrenocortical cell development, proliferation, and function, with aberrant cAMP-PKA signaling playing a significant role in the development of the majority of benign cortisol-producing ACTs. The role of abnormal cAMP-PKA signaling in adrenocortical tumorigenesis was first described in 1991 when early embryonic postzygotic somatic activating defects of the *GNAS* gene were implicated in the pathogenesis McCune-Albright syndrome (MAS). MAS manifests as café-au-lait skin macules, skeletal fibrous dysplasia, and multiple endocrinopathies including precocious puberty, testicular and thyroid lesions, phosphate wasting, growth hormone excess, and, rarely, neonatal hypercortisolism primarily due to bilateral adrenocortical hyperplasia. This form of BAH develops from adrenocortical cells with fetal features and is termed PBAD (2, 49). *GNAS* encodes the α-subunit of the stimulatory G protein (G_s_α). Mosaic gain-of-function pathogenic variants in *GNAS* cause constitutive activation of the cAMP-PKA pathway (4).

**Figure 4:**
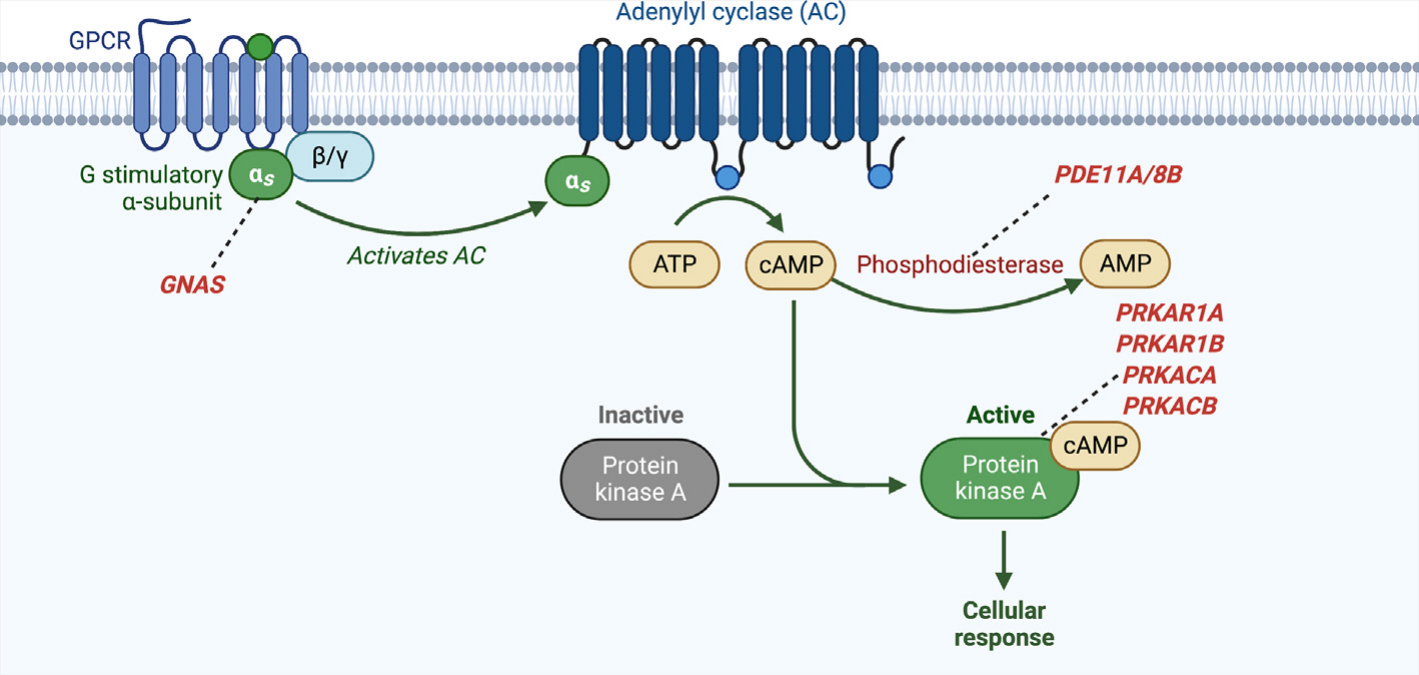
Activation of cyclic AMP pathway through various genetic defects in adrenocortical tumors and hyperplasias. *GNAS, gene coding for the stimulatory subunit α of the G-protein (Gsα); PDE8B, phosphodiesterase 8B gene; PDE11A, phosphodiesterase 11A gene; PRKACA, protein kinase, cAMP-dependent, catalytic, alpha; PRKAR1A, protein kinase, cAMP-dependent, regulatory, type I, α gene*.

PKA plays a role in the control of a variety of cellular processes including metabolism, transcription, cell cycle progression, and apoptosis. It is a ubiquitous cAMP-dependent serine-threonine kinase, with four isoforms for both its regulatory subunits (R1α, R1β, R2α, and R2β) and catalytic subunits (Cα, Cβ, Cγ, and PRKX), with each isoform having individual localization and specificity. The cAMP-PKA pathway is activated in the adrenocortical cell by the binding of ACTH to the ACTH receptor, a G-protein coupled receptor (GPCR) encoded by the *MC2R* gene. This leads to exchange of guanosine triphosphate (GTP) for guanosine diphosphate (GDP) on the α subunit of the associated heterotrimeric G protein and dissociation of the α subunit (encoded by *GNAS*) from the βγ dimer. The α subunit then binds to adenylyl cyclase (AC) with activation of its enzymatic activity and subsequent production of cAMP from adenosine triphosphate (ATP). cAMP then binds to the regulatory subunits (R) of PKA, which allows for the release of the catalytic subunits (C) of PKA. The catalytic subunits of PKA then mediate serine-threonine phosphorylation of target molecules, including the transcription factor CREB (cAMP response element-binding protein). Phosphodiesterases (PDEs) are the only known enzymes that degrade cyclic nucleotides and regulate the cAMP-PKA pathway through the regulation of cAMP levels (50) (**Figure 4**).

### Micronodoular Adrenocortical Hyperplasia

Micronodular BAH accounts for <2% of cases of endogenous CS and may be familial, and inherited in an autosomal dominant manner, or sporadic. It can be divided histologically into at least two subclasses: PPNAD and isolated micronodular adrenocortical disease (i-MAD). PPNAD is characterized by pigmented adrenocortical nodules with atrophic surrounding adrenocortical tissue and is more common than and more frequently familial than i-MAD, whereas i-MAD has limited or absent nodular pigment with hyperplasia in the surrounding zona fasciculata (51). Ninety percent of cases of PPNAD are associated with Carney complex (CNC) (c-PPNAD) though PPNAD may be isolated (i-PPNAD) (52, 53).

CNC is a rare, autosomal dominant, multiple endocrine neoplasia and lentiginosis syndrome comprised of abnormal cutaneous and mucosal pigmentation, myxomas predominantly of the heart, skin, and breast, psammomatous melanotic schwannomas, breast ductal adenomas, osteochondromyxomas, endocrine neoplasms, and other non-endocrine tumors. C-PPNAD is diagnosed in 25% to 60% of patients with CNC and is the most prevalent endocrine neoplasm in this syndrome, identified in almost all patients on autopsy. Pituitary adenomas or hyperplasia, thyroid tumors, and gonadal tumors make up the remaining endocrine tumors associated with CNC (54–56). CNC may be familial in up to 70% of cases and is predominantly caused by inactivating defects *PRKAR1A* (17q24.2–24.3 locus [CNC1 locus] of the long arm of chromosome 17), which encodes the regulatory subunit type 1α (R1α) of PKA. CNC was the first disease found to be the result of a genetic defect in a gene encoding a component of the PKA enzyme, with inactivating *PRKAR1A* defects causing loss of regulation of the catalytic subunits of PKA and constitutive activation of the cAMP-PKA pathway. Thirty-seven percent of patients with sporadic CNC and more than 70% of patients with familial CNC carry *PRKAR1A* pathogenic variants, with almost 100% penetrance (3, 52, 57). Data initially suggested that *PRKAR1A* functions as a “classic” tumor-suppressor gene, with loss of heterozygosity at the *PRKAR1A* locus in tumor tissue, however some data show that haploinsufficiency of *PRKAR1A* may be adequate for increased PKA activity and the early development of certain tumors (58, 59). Most pathogenic *PRKAR1A* variants lead to *PRKAR1A* haploinsufficiency due to mRNA nonsense-mediated decay of the mutant sequence, that leads to predicted absence of the mutant protein products in affected cells (52, 60). A second affected locus on chromosome 2p16 (CNC2 locus) was identified by genetic linkage analysis of tumors in most of the remaining patients with CNC that do not carry a germline *PRKAR1A* defect, though the responsible gene at this locus has not yet been identified (61, 62). In addition, copy number gains of the *PRKACB* gene locus, on chromosome 1, which encodes the catalytic subunit β (Cβ) of PKA, were identified in a patient with CNC presenting with abnormal skin pigmentation, myxomas, and acromegaly, though defects in this gene have not been associated with c-PPNAD (63). Possibly pathogenic germline *PRKACB* variants were also recently reported in two of 148 patients with PPNAD and related disorders that did not have other PKA-related defects. The first subject with the *PRKACB* gene variant (c.858_860GAA [p.K286del]) presented with short stature, multiple skeletal developmental malformations, and severe developmental delay suggesting a role for *PRKACB* defects in bone pathology, with functional studies demonstrating that this variant affected PRKACB protein stability and led to increased PKA signaling. The other subject carried the c.899C>T (p.T300M or p.T347M in another isoform) *PRKACB* variant and presented with a PPNAD-like phenotype without any other manifestations of CNC though functional studies demonstrated that this variant did not affect protein stability or response to cAMP and its pathogenicity remains uncertain (64). *PRKAR1A* defects have also been implicated in i-PPNAD, where there is a genotype-phenotype correlation. A study that included 353 subjects with germline *PRKAR1A* pathogenic variants or a diagnosis of CNC and/or PPNAD showed that among subjects with i-PPNAD and *PRKAR1A* defects the germline c.709–7del6 defect was more frequent whereas the remainder of these patients carried the p.Met1Val defect, and subjects less than 8 years of age rarely had *PRKAR1A* defects (52). Somatic defects in *PRKAR1A* have also been described in cortisol-producing ACTs, though less frequently, including CPAs and ACCs (65). ACC was also described in two patients with c-PPNAD due to germline *PRKAR1A* pathogenic variants (65, 66). ACTH-dependent CS due to an ACTH- producing pituitary adenoma has rarely been described in CNC (67, 68). Genetic alterations in *PRKAR1B*, which encodes the regulatory subunit 1β of PKA have been described in ACTs, with germline *PRKAR1B* variants identified in i-MAD and somatic *PRKAR1B* copy number gains (CNGs) found in CPAs. However, the contribution of genetic alterations in *PRKAR1B* to adrenocortical tumorigenesis may be different than those from defects affecting other subunits of PKA, as *PRKAR1B* variants and *PRKAR1B* CNGs led to decreased (rather than increased) overall PKA activity *in vitro* (69).

Genetic alterations in genes encoding cyclic nucleotide PDEs, that lead to aberrant cAMP-PKA signaling, have also been implicated in the development of micronodular BAH and other cortisol-producing ACTs. A single-nucleotide polymorphism-based genome-wide association study that included patients with i-MAD or i-PPNAD not caused by known genetic defects (defects in *GNAS* or *PRKAR1A*) showed that abnormalities in genetic loci harboring PDE genes were most likely to be associated with the disease. Inactivating defects *PDE11A*, which encodes phosphodiesterase type 11A, were the most frequently linked, followed by defects in *PDE8B*, which encodes phosphodiesterase type 8B (70). A higher frequency of *PDE11A* variants has also been found in patients with CNC, with *PDE11A* defects being associated with increased development of PPNAD and/or testicular large-cell calcifying Sertoli cell tumors (LCCSCT) in those with *PRKAR1A* defects, possibly acting as a genetic modifying factor in these patients (71). Heterozygous germline defects in *PDE11A* were also found to be more prevalent in patients with other ACTs, including ACC, CPAs, and PBMAH, when compared to age and/or sex-matched controls and were described in one patient with a non-secreting adrenocortical adenoma (72). Inactivating defects in *PDE11A* have also been implicated in the development of other tumors including prostate cancer and testicular germ cell tumors (71, 73, 74). A single germline *PDE8B* missense substitution was first described in a pediatric patient with i-MAD and CS. The patient’s father who harbored the same *PDE8B* defect, was not diagnosed with CS but did have elevated serum midnight cortisol (75). Subsequently in a case-control study of 216 unrelated patients with adrenocortical tumors (including PPNAD, PBMAH, CPAs, non-secreting adrenocortical tumors, and ACC) and 192 controls, nine different *PDE8B* sequence changes were found in the patients and controls with two variants that were identified only in the patient group demonstrating significant potential to impair protein function *in vitro* and *in silico* (76).

Defects in genes encoding the catalytic subunits of PKA that lead to increased PKA activity have also been found to play a role the pathogenesis of micronodular BAH. Germline copy number gains resulting in amplification of *PRKACA*, the gene that encodes the catalytic subunit (Cα) of PKA, have been implicated in the development of i-MAD. Three patients with sporadic i-MAD and 2 patients with familial PBMAH were the first patients found to harbor such germline copy number gains of the genomic region on chromosome 19p which includes the entire *PRKACA* gene (1, 77).

Finally, micronodular BAH has also been associated with abnormalities of the *Wnt*–β-catenin pathway, which may act as a genetic modifier. One study identified somatic defects in *CTNNB1* in 11% of patients with PPNAD (a germline *PRKAR1A* defect was identified in 1 of the 2 patients with somatic *CTNNB1* defects). These *CTNNB1* defects occurred in relatively large adrenocortical adenomas that developed in the background of PPNAD and were not found in the surrounding hyperplastic adrenocortical tissue (78). In a second study that included tissue from nine subjects with PPNAD (with eight of the nine harboring *PRKAR1A* defects), including five with macronodules, activating somatic *CTNNB1* defects were identified in two of the five macronodules but not in the micronodules or in the contralateral adrenal gland, whereas all PPNAD tissues had β-catenin accumulation including within the macronodules, micronodules, and internodular tissue (79).

### Macronodular Adrenocortical Hyperplasia

PBMAH is a rare cause of adrenal CS, accounting for <2% of cases, and is mostly isolated or sporadic, and inherited in an autosomal dominant fashion when hereditary. Adrenal cortisol secretion in this disease was initially considered to be ACTH-independent and thus this form of BAH was formerly called ACTH-independent macronodular adrenal hyperplasia (AIMAH); however, studies have since demonstrated that ACTH secretion from clusters of adrenocortical cells in PBMAH may in part regulate cortisol secretion by paracrine action (80). PBMAH has also previously been termed bilateral macronodular adrenal hyperplasia (BMAH), primary macronodular adrenal hyperplasia (PMAH), massive macronodular adrenocortical disease (MMAD), autonomous macronodular adrenal hyperplasia (AMAH), ACTH-independent massive bilateral adrenal disease (AIMBAD), and “giant” or “huge” macronodular adrenal disease. Histologically, PBMAH can be subclassified into type I PBMAH which is characterized by internodular atrophy, and the more common type II PBMAH, which is diffusely hyperplastic without residual normal or atrophic internodular tissue (81). Adrenocortical cells in PBMAH express aberrant (ectopic or excessive) hormone receptors in 77% to 87% of cases whereas such aberrant receptor expression has been found less frequently in adrenocortical adenomas and ACC (81). These aberrant receptors are members of the GPCR family and are linked to steroidogenesis. Such receptors include those for glucose-dependent insulinotropic peptide (GIP) (implicated in food-dependent CS), vasopressin, β-adrenergic agonists, luteinizing hormone/choriogonadotropin (LH/hCG), serotonin, angiotensin II, and glucagon (82–92). The underlying genetic changes leading to this ectopic receptor expression have not yet fully been described. Food dependent CS is a rare subtype of macronodular BAH associated with ectopic expression of the GIP receptor (GIPR). The molecular pathogenesis of ectopic GIPR expression in this disease was investigated in a study that included adrenal tissue from 15 ACTs including CPAs and macronodular BAH. This study showed that GIPR expression occurred due somatic duplications in chromosome region 19q13.32, that contains the GIPR locus, with resulting transcriptional activation of a single allele of the GIPR gene in three of the ACTs (2 CPAs and 1 macronodular BAH). In the CPAs, the duplicated 19q13.32 region was rearranged with other chromosome regions however these chromosome rearrangements did not result in gene fusion but instead placed the GIPR gene in a genomic environment near cis-acting regulatory regions favoring transcriptional activation. In the macronodular BAH sample, a duplication of 19q without chromosome rearrangement was identified (93).

The bilateral nature PBMAH as well as the cases of familial PBMAH and the association of PBMAH with familial tumor syndromes suggested that this disease could be caused by underlying germline genetic defect(s). In 2013, germline inactivating defects in *ARMC5* were linked to this disease when genotyping of 33 patients with PBMAH showed defects in *ARMC5* in 55% (18/33) of these patients (5). In subsequent studies, the prevalence of *ARMC5* defects in patients with PBMAH has been estimated to be 21% to 26% (94–96) *ARMC5* is a tumor suppressor gene which encodes a cytosolic protein without enzymatic activity that has an armadillo repeat domain, similar to the gene for β-catenin that also contains armadillo repeats (97, 98). In this initial study of 33 patients with PBMAH, functional studies showed that inactivation of *ARMC5* led to reduced expression of steroidogenic enzymes and *MC2R* with abnormal cortisol production. A gradual process of adrenocortical cell dedifferentiation and growth of bilateral masses was evident in these patients, with the hypercortisolemia being more likely a result of the increased adrenocortical mass than cortisol overproduction. Enlargement of the adrenal glands may be due to loss of the ability to induce apoptosis in adrenocortical cells with *ARMC5* defects, as shown experimentally in human adrenocortical cell lines when compared to wild-type cell lines (5, 94). Studies in *Armc5* knockout mice have shown that this gene may play a significant role in in fetal development, T-cell function, and adrenal gland growth homeostasis. *Armc5* haploinsufficiency in these mice manifests as CS later in life, with implication of both the cAMP-PKA and the *Wnt*-β−catenin pathways (97, 99). *ARMC5* variants may act as genetic modifiers in PPNAD due to a *PRKAR1A* defect, and may affect the presence and severity of hypercortisolemia in patients harboring these variants (100). In addition, in 2015 an association between *ARMC5* defects and primary aldosteronism was first described (22). *ARMC5* defects have also been linked to the development of meningiomas, as shown in one family with meningioma and adrenal hyperplasia with *ARMC5* loss of heterozygosity in the meningioma DNA (101).

Rarely, PBMAH is a component of autosomal dominant multiple tumor syndromes including familial adenomatous polyposis (FAP), multiple endocrine neoplasia type 1 (MEN1), or hereditary leiomyomatosis and renal cell carcinoma (HLRCC) (102–104). The causative genetic defects in these familial tumor syndromes were the only genetic defects associated with PBMAH until inactivating defects of *ARMC5* were found to cause this disease (5). Germline inactivating defects in the tumor suppressor gene *adenomatous polyposis coli* (*APC*), which encodes the APC protein, cause FAP. The APC protein is a component of the β-catenin Axin degradation complex which negatively regulates the *Wnt-*β-catenin signaling pathway, with biallelic *APC* inactivation leading to transcriptional activation of the *Wnt*-signaling pathway. Classic FAP is comprised of multiple colorectal adenomas (>100) that predispose to colorectal cancer, fundic gland polyps, and duodenal adenomas that predispose to duodenal cancer. FAP may also have extraintestinal manifestations such as follicular or papillary thyroid cancer, childhood hepatoblastoma, central nervous system tumors, desmoid tumors, sebaceous or epidermoid cysts, lipomas, osteomas, fibromas, supernumerary teeth, and juvenile nasopharyngeal angiofibromas. FAP has also been associated with ACTs including PBMAH, adrenocortical adenomas, and ACC. In one cohort, 16% of patients had adrenal masses of which 97% were benign and 80% were adenomas, with 23% of the adrenal masses being bilateral. At diagnosis, the median diameter of these adrenal masses was 1.7 cm (interquartile range (IQR) 1.4–3.0) with median maximal diameter of 2.5 cm (IQR 1.7–4.1) (105).

MEN1 is caused by inactivating defects in *MEN1*, a tumor suppressor gene located at the 11q13 locus (106, 107). *MEN1* encodes the protein menin, whose exact role in tumorigenesis is yet to be identified, though it has been implicated in the regulation of transcription, genome stability, cell division, and cell proliferation (106–109). This syndrome leads to a predisposition to a multitude of endocrine neoplasms predominantly of parathyroid, enteropancreatic, and anterior pituitary origin with other endocrine tumors including foregut carcinoid tumors, ACTs, and rarely pheochromocytoma. Nonendocrine tumors associated with MEN1 include meningiomas and ependymomas, lipomas, angiofibromas, collagenomas, and leiomyomas (110). In a retrospective cohort study of 715 patients with MEN1, 20.4% had adrenal enlargement. Adrenal tumors greater than 1 centimeter were described in 58.1% of these cases with bilateral tumors being present in 12.5% of cases. 15.3% of patients had hormonal hypersecretion, which was found only in patients with ACTs. When compared to controls with adrenal incidentalomas, MEN1-related adrenal tumors exhibited more cases of primary aldosteronism and ACC (102).

HLRCC is a syndrome caused by germline defects in *fumarate hydratase* (*FH*), a possible tumor suppressor gene (111). *FH* encodes fumarate hydratase, an enzyme that is a component of the mitochondrial Krebs or tricarboxylic acid cycle. Defects in *FH* may lead to HLRCC through increased cellular dependence on glycolysis and pseudohypoxia, though the molecular pathogenesis of HLRCC has not been completely elucidated (112, 113). Patients with HLRCC develop cutaneous and uterine leiomyomas (rarely leiomyosarcomas) and renal cell carcinoma. Approximately 7.8% of patients with HLRCC develop ACTs including PBMAH and adrenocortical adenomas that may be cortisol-producing or non-functional (104, 114). Germline *FH* pathogenic variants have also been implicated in the development of pheochromocytomas and paragangliomas (115, 116).

The development of PBMAH has also been linked to genetic defects that lead to aberrant cAMP-PKA signaling. Such defects include inactivating germline *PDE11A* variants that are found in 24% to 28%, of cases of PBMAH, as well as inactivating germline *PDE8B* defects, activating somatic *GNAS* defects without MAS, and *PRKACA* copy number gains (72, 76, 81, 117). Another subtype of macronodular BAH, PBAD, has been described in patients with MAS and CS (2, 118). *PRKAR1A* defects have not been identified in PBMAH, however somatic losses of the 17q22–24 region in PBMAH lead to PKA subunit and enzymatic activity changes and altered PKA signaling that is similar to that of other adrenal tumors with *PRKAR1A* defects or 17q losses (119). A single case of PBMAH caused by two defects in the same allele of *MC2R* which encodes the ACTH or melanocortin 2 receptor has been described. The presence of both of these defects (p.C21R and p.S247G defects) in the same molecule led to constitutive activity of the receptor, with the co-expression of the normal *MC2R* allele leading to retention of a normal response to ACTH. These defects ultimately resulted in abnormal activation of the cAMP-PKA pathway and clinical hypersensitivity to ACTH, though either defect alone would have produced an inactive receptor (120). Additionally, the role of the cAMP-PKA pathway in the pathogenesis of PBMAH is demonstrated in cases of PBMAH with aberrant receptor expression as the majority of these receptors are GPCRs that stimulate AC, with resulting increased cAMP-PKA signaling.

Additional possible genetic alterations including somatic defects in *DOTIL*, which encodes a histone H3 lysine methyltransferase, and *HDAC9*, which encodes a histone deacetylase, have been reported in a small number of patients with PBMAH. Both of these genes play a role in histone modification, chromatin organization and modification of gene transcription. A defect in another gene, the *Endothelin receptor type A (EDNRA)* gene, which encodes a G-coupled protein, was found in adrenal tissue from two siblings from a family with familial PBMAH (121), but its contribution to adrenocortical tumor development remains questionable.

### Cortisol-Producing Adrenocortical Adenomas

As in BAH, the cAMP-PKA pathway also plays an important role in the development of CPAs. However in contrast to BAH where germline defects leading to aberrant cAMP-PKA signaling are most prominent, somatic genetic alterations affecting this pathway predominate in CPAs (122). In 2013, whole exome sequencing of tumor-tissue specimens from patients with unilateral CPAs identified somatic *PRKACA* in 8 out of 10 adenomas, with additional sequencing of another 129 adenomas demonstrating a p.Leu206Arg variant in 14 of these 129 adrenocortical adenomas. Defects in *PRKACA* were associated with a more severe phenotype and were found only in patients with overt CS (1). Another 3 studies from China, Japan, and the United States subsequently showed similar findings, with *PRKACA* defects found in 42% of patients with CPAs with overt CS (123). Activating *PRKACA* defects lead to constitutive activation of PKA by abolishing the interaction between the regulatory and catalytic subunits of PKA and may also modify substrate specificity with hyperphosphorylation of certain PKA substrates (124). Recently, an activating somatic defect in *PRKACB* was also described in a patient with a CPA,with increased sensitivity to cAMP demonstrated in *in vitro* studies (125). *PRKACA* defects have also been found in cardiac myxomas, and chromosomal *PRKACA* rearrangements were identified in fibrolamellar hepatocellular carcinoma and in intraductal oncocytic papillary neoplasms of the pancreas and bile duct, along with *PRKACB* defects (126–128). Inactivating somatic defects in *PRKAR1A* as well as activating somatic defects in *GNAS* have also been described in CPAs with a prevalence of 5%, and 4.5% to 11%, respectively (60, 65). Both *PRKAR1A* and *GNAS* defects can cause increased cAMP-PKA signaling, however a whole genome expression profile study revealed that not all cAMP activation is the same. In this study, overexpression of the MAPK and p53 signaling pathways was demonstrated in adrenal lesions with both *PRKAR1A* or *GNAS* defects, however *PRKAR1A-*mutant tissues overexpressed genes related to the *Wnt*-signaling pathway (*CCND1*, *CTNNB1*, *LEF1*, *LRP5*, *WISP1*, and *WNT3*), whereas *GNAS-*mutant tissues showed increased expression of genes involved in extracellular matrix receptor interaction and focal adhesion pathways (*NFKB, NFKBIA*, and *TNFRSF1A*). CPAs without defects in *GNAS*, *PRKAR1A*, *PDE11A*, or *PDE8B* were also found to have abnormalities in the cAMP-signaling pathway with variant-negative CPAs having significantly decreased PDE activity (129).

## Conclusion

Significant advances have been made in understanding the genomic underpinnings of PA and CS in recent years. The use of genomic tools and next generation sequencing have allowed for the discovery that aberrant intracellular calcium signaling plays an integral role in the development of PA and that abnormalities in the cAMP-PKA pathway and/or *ARMC5* are central in the development of benign cortisol-producing tumors. The role of abnormal Wnt-β-catenin signaling in ACT development has also been highlighted. These findings have built a foundation for the discovery of more targeted diagnostic, therapeutic, and prognostic tools that may lead to less invasive diagnostic and therapeutic methods, as well as for the development of future gene-based tumor classifications that will allow improved genetic counseling and screening for familial cases, and better prognosis.

## Author Contributions

All authors listed have made a substantial, direct, and intellectual contribution to the work and approved it for publication.

## Funding

This work was supported by the research project Z01-HD008920 (Principal Investigator: CS) of the Intramural Research Program of the *Eunice Kennedy Shriver* National Institute of Child Health & Human Development (NICHD), National Institutes of Health (NIH), Bethesda, MD, USA.

## Conflict of Interest

CS holds patents on the *PRKAR1A*, *PDE11A*, and *GPR101* genes and/or their function and has received research funding from Pfizer Inc. on the genetics and treatment of abnormalities of growth hormone secretion.

The remaining authors declare that the research was conducted in the absence of any commercial or financial relationships that could be construed as a potential conflict of interest.
